# Endoscopic recurrent laryngeal neuropathy grade prevalence in a sample of thoroughbred yearlings at public auction in South Africa (2013–2019)

**DOI:** 10.4102/jsava.v91i0.2013

**Published:** 2020-04-20

**Authors:** Sean M. Miller

**Affiliations:** 1 Summerveld Equine Hospital, Summerveld, South Africa

**Keywords:** laryngeal function, recurrent laryngeal neuropathy, equine, larynx, endoscopy

## Abstract

Endoscopy of thoroughbred (TB) yearlings at public auctions is common in South Africa. Laryngeal function (LF) is a common concern of buyers of young TBs. Cancellation of sale because of LF abnormalities is a concern for both the vendor and the buyer, with recurrent laryngeal neuropathy (RLN) being a common cause of sale cancellation. The aim of this descriptive study was to determine the prevalence of RLN at South African premier TB yearling sales. This study was designed as a retrospective descriptive analysis of upper respiratory tract (URT) endoscopic examinations to determine RLN grade, performed at two premier TB yearling sales in South Africa. Results of buyer-requested endoscopic examination from 2013 to 2019 were included. Results from the yearling sales were analysed for prevalence of RLN grade (using Rakestraw’s 4-point system) and compared to similar previously published studies. For analysis of effects of gender on RLN grading, horses were grouped and Fisher’s exact test was used to determine if there was a relationship between gender and grade. For comparison of the effects of age on grade, and sales year on grade, a Kruskal–Wallis test was conducted. A value of *p* < 0.05 was considered significant. A total of 858 horses were examined out of 4149 offered for sale; there were 57.58% colts and 42.42% fillies (mean age of 18.1 months). The annual percentage for grade 1 was 84.04% ± 9.98%, for grade 2: 14.49% ± 10.69%, for grade 3: 0.71% ± 0.57% and for grade 4: 0.76% ± 0.94%. There were no other significant findings. The exclusive nature of the sale and the increasing proclivity for pre-sale scoping may have skewed the results. This study shows that RLN grade incidences in TB yearlings at public auctions in South Africa are as follows: grade 1: 84.04%, grade 2: 14.49%, grade 3: 0.71% and grade 4: 0.76%. The results were similar to that of an adult population of horses examined in South Africa in a previous study.

## Introduction

Endoscopy of thoroughbred (TB) yearlings as part of the yearling sale procedure is common in South Africa. Laryngeal function (LF) is a common concern for potential buyers of young TBs worldwide. The main concern for buyers is to identify abnormal function of an arytenoid (usually the left) before conclusion of the sale, allowing the buyer to cancel the sale and avoid purchasing a horse with recurrent laryngeal neuropathy (RLN), avoiding potential poor performance and obviating the need for future surgical intervention. This concern is justified as it has been shown that TB yearlings with grades 1 and 2 RLN showed significantly better racing performance as adults compared to yearlings with grade 3 RLN (Stick et al. 2001:962–967).

There are multiple problems that can affect RLN. One of the most common problems presents as laryngeal hemiplegia. This is the loss of abduction of the arytenoid cartilage, most commonly the left. Loss of abduction and the resultant increased negative pressure in the laryngeal lumen during exercise result in inward collapse of the arytenoid, leading to increasing obstruction of the upper respiratory tract (URT), and can lead to poor performance (Kelly 2016).

In a study conducted in the United Kingdom (UK) comprising 197 TB foals, the prevalence of grade 3 or 4 laryngeal asymmetry was 19%, and when 187 of these were re-examined as yearlings, 23% were grade 3 or 4 (Lane 2004:31–32). Studies showing comparative prevalence of RLN grading are shown in Table 1. The reasons for the wide variation seen in the prevalence in each study may be attributed to a number of factors including the life stage when the horses were examined (yearlings vs. adults), exercise stage (at rest or immediately post-racing), differences in the observers’ definition of arytenoid asymmetry, experience of the observer, selection criteria for endoscopic examination and the grading scale used.

**TABLE 1:** Comparison of previous similar studies performed.

Author	Location	Horses	Grade (%)			
			1	2	3	4
Stick et al. 2001	US	TB yearlings	35.0	40.0	22.0	3.0
Garrett et al. 2010	US	Yearlings at auction	19.0	79.0	2.0	0.0
Brown et al. 2005	Australia	Competing racehorses	-	1.3	1.3	0.0
Anderson, Kannegieter & Goulden 1997	NZ	Young racehorses	52.0	33.0	14.0	1.0
Pascoe et al. 1981; Raphel 1982; Sweeny, Mason & Soma 1991	North America	Race horses	-	3.8	3.8	1.3–3.3

US, United States of America, NZ, New Zealand; TB, thoroughbred.

In a retrospective dynamic over-ground endoscopy study in South Africa, abnormal arytenoid function during exercise, consistent with laryngeal hemiplegia, was detected in 33% of the TB race horses (Mirazo et al. 2014). However, this study included only those horses specifically referred for further diagnosis of poor performance or respiratory noise and, as such, the prevalence of detectable problems may be higher than in the general population. The study compares well with other studies involving TB racehorses which mainly included those referred for investigation of respiratory noise and/or poor performance of approximately 8% – 40% of horses (Allen & Franklin 2010:186–191; Barakzai & Dixon 2011:18–23; Dart et al. 2001:109–112; Desmaizieres et al. 2009:347–352; Tan, Dowling & Dart 2005:243–248).

In another South African cross-sectional study looking at post-racing prevalence of URT disorders in horses 2 to 9 years old (mean: 4 years), RLN was found at a prevalence of grade 1 in 96%, grade 2 in 1.8%, grade 3 in 0.4% and grade 4 in 0.6% of TB race horses (Saulez & Gummow 2009:431–435). This study compares favourably with the Australian report that used similar cross-sectional inclusion criteria and grading scale for RLN and also evaluated racehorses soon after racing (Brown et al. 2005:397–401).

During South African yearling sales, the horses are endoscopically examined post-sale before release from the sales grounds by a veterinarian appointed by the buyer. Any horse having been found to have a number of URT abnormalities is liable to ‘fail’ the URT endoscopic examination and thus the sale is cancelled. This differs from a number of international sales where endoscopic examination is requested by the sales company, such as New Zealand Bloodstock (Kelly 2016). A decision regarding the grading is made by an expert panel where video endoscopic examination is performed and independent assessments are made, followed by a consensus decision. In other countries, such as the UK and Ireland, horses are lunged by the sales company and only horses making an abnormal respiratory noise are endoscopically examined (Kelly 2016).

The aim of this descriptive study was to determine the prevalence of RLN grades at South African premier TB yearling sales. To the author’s knowledge, RLN in South African TB premier yearling sales has not been investigated or previously reported.

## Study design and case selection

This study was designed as a retrospective analysis of URT endoscopic examinations to determine RLN grade, performed at two premier TB yearling sales (2013–2019) held in South Africa annually. Results of endoscopic examinations performed on behalf of buyers for one particular equine veterinary practice for sales from 2013 to 2019 for the first premier sale and from 2016 to 2019 for the second premier sale were included in the study. A total of 858 horses out of 4149 (20.68%) offered for sale were examined.

Cases included were all TB yearlings offered at public auctions that were requested to be examined by the purchaser. Records of the results of these examinations were used to obtain the data. Cases were excluded if only a repository examination was available. Cases with right-sided RLN abnormalities were excluded. Horses with abnormalities other than left RLN abnormalities were also excluded.

## Materials and methods

All of the examinations in the present study were conducted by the researcher who is an experienced race horse veterinarian (S.M.M.). The URT was examined as follows: the horses were restrained with a head collar with the handler and the veterinarian standing on the horse’s left, a twitch was applied to the upper lip and the endoscope was passed up the right ventral meatus. Sedation was not used because of its effect on RLN grading (Ducharme et al. 1991:180–184). For all horses, either both nostrils were occluded and released, or swallowing was stimulated by touching the endoscope to the arytenoid cartilage and was continued until sufficient swallowing cycles were observed for a grade to be determined. For horses graded 3 or 4, an additional independent experienced veterinarian or South African qualified specialist equine surgeon appointed by the auctioneers examined the horse to confirm the grading. This second examination was conducted as soon as possible after the first examination, under the same conditions and in the same manner. The second examining veterinarians grading was used to confirm grades 3 and 4 RLN as per the sales requirements and this confirmatory grade was the final grade used for these horses in this study. Grades 1 and 2 horses were not subjected to a second examination.

Grading of RLN used a 4-point grading system (Table 2) (Rakestraw et al. 1991:122–127; Saulez & Gummow 2009:431–435). The grading system was used at sales as established by the auctioneers in South Africa, who consider grades 1 and 2 as ‘pass’ and grade 3 or 4 as ‘fail’, irrespective of subgrades, as outlined in the sales agreement.

**TABLE 2:** Grading of recurrent laryngeal neuropathy.

Grade	Description
1	Synchronous full abduction of both arytenoid cartilages during inspiration or breath holding, or after swallowing
2	Asynchronous full abduction of the left arytenoid cartilage (hesitation, flutter or delay) can be achieved and maintained during inspiration or breath holding, or after swallowing
3	Asynchronous abduction of the left arytenoid cartilage (hesitation, flutter or delay). Substantial movement is present, but full abduction cannot be achieved and maintained during inspiration or breath holding, or after swallowing
4	No appreciable abduction of the left arytenoid cartilage during any phase of respiration

*Source*: Rakestraw, P.C., Hackett, R.P., Ducharme, N.G., Nielan, G.J. & Erb, H.N., 1991, ‘Arytenoid cartilage movement in resting and exercising horses’, *Veterinary Surgery* 20(2), 122–127. https://doi.org/10.1111/j.1532-950X.1991.tb00319.x; Saulez, M.N. & Gummow, B., 2009, ‘Prevalence of pharyngeal, laryngeal and tracheal disorders in thoroughbred racehorses, and effect on performance’, *Veterinary Record* 165(15), 431–435. https://doi.org/10.1136/vr.165.15.431

The results were tabulated using an Excel spreadsheet (Microsoft Excel 2007, Microsoft Corp, Redmond, WA, United States). The results were expressed as a prevalence for each grade. For analysis of the effects of gender on RLN grading, horses with grades 1 and 2 were considered ‘normal’ and those with grades 3 and 4 were considered ‘abnormal’. Fisher’s exact test (Fisher 1922:87–94) was used to determine if there was a relationship between gender and grade, because of one or more instances of the expected value being less than 5. For comparison of the effects of age on grade, and sales year on grade, a Kruskal–Wallis test was conducted (Gibbons 1976). For all comparisons, a value of *p* < 0.05 was considered significant. Statistical analysis was performed by the researcher using IBM SPSS Statistics for Windows, Version 25.0 (IBM Corp., Armonk, NY, United States).

### Ethical considerations

The data was collected using standard or accepted veterinary procedures during routine examinations.

## Results

A total of 858 horses out of the 4149 offered for sale over the 7-year period (20.68%) were examined. The horses endoscopically examined per sale ranged between 13.71% and 26.85%. There were 494 colts (57.58%) and 364 fillies (42.42%) examined, with a mean age of 18.1 months (range 13–26 months) at the time of examination from 2013 to 2019. The mean percentage ± standard deviation for grade 1 was 84.04% ± 9.98%, for grade 2: 14.49% ± 10.69%, for grade 3: 0.71% ± 0.57% and for grade 4: 0.76% ± 0.94% (Table 3).

**TABLE 3:** Endoscopic examination grading of recurrent laryngeal neuropathy of yearlings at South African sales from 2013 to 2019.

Grade	2013		2014		2015		2016		2017		2018		2019		% Mean ± SD
	*n*	%	*n*	%	*n*	%	*n*	%	*n*	%	*n*	%	*n*	%	
Grade 1	65	95.96	81	78.64	67	83.75	119	85.61	133	92.36	142	87.12	105	65.22	84.04 ± 9.98
Grade 2	1	1.47	21	20.39	10	12.5	19	13.67	10	6.94	19	11.66	56	34.78	14.49 ± 10.69
Grade 3	1	1.47	1	0.9	1	1.25	0	0	1	0.69	1	0.61	0	0	0.71 ± 0.57
Grade 4	1	1.47	0	0	2	2.5	1	0.72	0	0	1	0.61	0	0	0.76 ± 0.94
**Total horses**	**68**	**-**	**103**	**-**	**80**	**-**	**139**	**-**	**144**	**-**	**163**	**-**	**161**	**-**	**-**

*n*, Number of individuals; %, percentage; SD, standard deviation.

Analysis of the effects for gender on RLN grading showed no significant difference between colts and fillies with grades 1 and 2 or with grades 3 and 4 RLN (Table 4). There was also no significant effect of age on grade. There was no significant difference between grades when compared by sales year.

**TABLE 4:** Gender versus recurrent laryngeal neuropathy grade in the present study.

Gender	Grade 1	Grade 2	Grade 3	Grade 4
Colts	407	80	3	4
Fillies	305	56	2	1
**Total**	**712**	**136**	**5**	**5**

## Discussion

The results of this study are, surprisingly, highly comparable to that of previous studies on older racehorses within South Africa (Saulez & Gummow 2009:431–435), with the only major difference being the percentage of grade 2 horses (14.49% compared to 1.8%). The reason for this may be that as horses age, the wastage rate for horses graded 2–4 would be high, with horses being retired or sent to stud. This leads to the assumption that horses with higher grades, still racing, are able to perform better irrespective of their RLN grade. The findings of the current study are mostly similar to that of an Australian study conducted on competing races horses, with the grade 2 RLN being the most dissimilar category (14.49% grade 2 to 1.3% for combined grades 2 and 3 [Brown et al. 2005:397–401]). The values for grade 4 for the New Zealand and North American studies of yearlings and young horses are fairly comparable to each other (0.71% vs. 1%, 0% and 3% [Anderson et al. 1997:188–192; Garrett et al. 2010:669–673; Stick et al. 2001:962–967]); however, the other grades vary greatly.

The reasons for these discrepancies may be multifactorial. Although the studies most similar to the current yearling study involved adult horses, this may be because of a similar definition of RLN used by the studies. Examination at a different life stage may play a part because laryngeal hemiplegia is progressive (Embertson 2004:42–44). This may account for the larger grade 2 groups in other older populations studied. There is also debate over the repeatability of resting endoscopy, suggesting that there is moderate variation in repetitive grading, which may also influence the results of different groups and that single day grading should be cautiously interpreted (Perkins et al. 2009:342–346; Pollock et al. 2009:354–360).

In the South African context, there has been a large push from both race horse veterinarians and stud farms to perform endoscopic examinations (often more than one per horse) before a yearling is put up for public auction, particularly at more prestigious sales. This is both an effort to prevent horses with RLN deficits from entering the auction to reduce the loss and a disappointment to both the stud farm and the potential buyer on auction day. The current study was conducted at the two most prestigious yearling sales in South Africa. So, while this study compares well with other studies on older horses, this may be because horses with RLN deficits have been removed from the population group before examination at these yearling sales. This would mean that the current study is only relevant to the sales population and may not be the same in the general racing population as horses in the general racing population may have bypassed the sales altogether or been obtained on other sales. The prevalence similar to Saulez and Gummow’s (2009:431–435) study, however, suggests that it would hold true for the older racing population in South Africa.

This study was limited because of the nature of the yearling sale in that the horses presented for endoscopy may be more valuable horses and horses not presented for post-sale endoscopy were considered ‘cheaper’ by the buyer and not worth the examination even though these were prestigious sales. Horses that failed endoscopic examination before the sale would not be present at the sale. Therefore, this sample may not be a representative sample of the TB racehorse population in South Africa. The large variation of grade 2 horses from year to year may be because of the sample size for each year, with 2013 having only 13.71% of the horses offered for purchase being examined versus 2019 where 26.85% of the horses offered for purchase were examined. The larger sample size in 2019 may have been a more representative sample than the sample of 2013.

It is ultimately important for both buyers and sellers of yearlings to be aware that a single endoscopic examination may give a skewed impression of a yearling’s RLN and serial examinations are more likely to yield an accurate result. They also need to be aware that RLN is progressive and may occur in the career of any race horse. In addition, there is some evidence that RLN is heritable and early recognition may be important in future breeding planning with affected and non-affected individuals (Gerber et al. 2015:390–397).

In conclusion, this study shows that RLN grading occurs at the following rates in TB yearlings at premier public auctions in South Africa: grade 1: 84.04%, grade 2: 14.49%, grade 3: 0.71% and grade 4: 0.76%. The results are similar to that of an adult population of actively racing horses examined in South Africa.
